# Early assessment of infarct size by feature tracking cardiac MRI in patients with myocardial infarction

**DOI:** 10.1186/1532-429X-15-S1-P196

**Published:** 2013-01-30

**Authors:** Sebastian Buss, Birgit Krautz, Nina P Hofmann, Kristin Breuninger, Yannick Sander, Rebekka Kammerer, Philipp Matheis, Lukas Rust, Christian Galuschky, Philip Raake, Sven T Pleger, Grigorios Korosoglou

**Affiliations:** 1 University of Heidelberg, Heidelberg, Germany; 2 TomTec Imaging Systems, Munich, Germany

## Background

The degree and quantification of contractile dysfunction and myocardial scar in patients after acute myocardial infarction (AMI) has important prognostic implications. Myocardial deformation parameters like strain and strain rate have been shown to be more sensitive markers for contractile dysfunction than standard 2-D parameters.

We sought to investigate whether strain and strain rate imaging, assessed by a novel non-invasive post-processing feature tracking algorithm (FTI) on pre-acquired regular CMR SSFP images, would allow quantification of regional left ventricular (LV) function and its relation to degrees of infarct trans-murality in patients after AMI.

## Methods

Cardiac magnetic resonance (CMR) imaging was performed on a 1.5T whole body MRI scanner (Philips Achieva) in 44 patients (mean age: 56 ± 12) 3 ± 1 days after successfully reperfused first ST segment elevation AMI. Peak systolic circumferential strain and strain rate were measured on standard CMR SSFP cine sequences and afterwards related to the trans-mural extent of myocardial infarction determined by contrast enhanced CMR (CE-CMR) 10 minutes after i.v. injection of 0.2mmol/kg Gd-DTPA (Magnevist). The feature tracking algorithm (2D CPA MR©, TomTec Imaging Systems GmbH) is a two dimensional deformation analysis of the myocardium that was originally designed for echocardiographic image analysis, which has now been transferred to CMR SSFP sequences without the need for additional scans.

## Results

Cut-off peak systolic circumferential strain values of -21% differentiated patients with global infarct sizes ≥20g from patients with smaller infarct sizes providing sensitivities and specificities of 94% and 74%. An infarcted myocardium with a segmental infarct size of ≥50% was differentiated from non-transmural infarcted segments with a cut-off peak systolic circumferential strain value of >-14% with a sensitivity of 92% and a specificity of 86%. Peak negative circumferential strain correlated closely with the quantitatively determined infarct size on a global (r=0.78, p<0.001) and segmental level (r=0.76, p<0.001).

## Conclusions

FTI provides a rapid and objective quantification of global and regional myocardial function and allows the discrimination between different degrees of trans-murality in patients after AMI.

## Funding

none

